# Evoked EMG *versus* Muscle Torque during Fatiguing Functional Electrical Stimulation-Evoked Muscle Contractions and Short-Term Recovery in Individuals with Spinal Cord Injury

**DOI:** 10.3390/s141222907

**Published:** 2014-12-03

**Authors:** Eduardo H. Estigoni, Che Fornusek, Nur Azah Hamzaid, Nazirah Hasnan, Richard M. Smith, Glen M. Davis

**Affiliations:** 1 Clinical Exercise and Rehabilitation Unit, Exercise Health and Performance, Faculty of Health Sciences, University of Sydney, Lidcombe, 2006 NSW, Australia; E-Mails: ehestigoni@yahoo.com.br (E.H.E.); che.fornusek@sydney.edu.au (C.F.); nazirah@um.edu.my (N.H.); richard.smith@sydney.edu.au (R.M.S.); glen.davis@sydney.edu.au (G.M.D.); 2 Department of Biomedical Engineering, Faculty of Engineering, University of Malaya, Kuala Lumpur 50603, Malaysia; 3 Department of Rehabilitation Medicine, Faculty of Medicine, University of Malaya, Kuala Lumpur 50603, Malaysia

**Keywords:** evoked EMG, fatigue, torque, FES contraction, spinal cord injured

## Abstract

This study investigated whether the relationship between muscle torque and m-waves remained constant after short recovery periods, between repeated intervals of isometric muscle contractions induced by functional electrical stimulation (FES). Eight subjects with spinal cord injury (SCI) were recruited for the study. All subjects had their quadriceps muscles group stimulated during three sessions of isometric contractions separated by 5 min of recovery. The evoked-electromyographic (eEMG) signals, as well as the produced torque, were synchronously acquired during the contractions and during short FES bursts applied during the recovery intervals. All analysed m-wave variables changed progressively throughout the three contractions, even though the same muscle torque was generated. The peak to peak amplitude (PtpA), and the m-wave area (Area) were significantly increased, while the time between the stimulus artefact and the positive peak (PosT) were substantially reduced when the muscles became fatigued. In addition, all m-wave variables recovered faster and to a greater extent than did torque after the recovery intervals. We concluded that rapid recovery intervals between FES-evoked exercise sessions can radically interfere in the use of m-waves as a proxy for torque estimation in individuals with SCI. This needs to be further investigated, in addition to seeking a better understanding of the mechanisms of muscle fatigue and recovery.

## 1. Introduction

With improved medical treatment, more people recover from spinal cord injury (SCI), with reduced degree of impairment. Nevertheless, the majority of individuals with SCI still present with significant muscle paresis [1]. In those with intact lower motor neurones and muscles innervation below the level of injury Functional Electrical Stimulation (FES)-evoked exercise can increase muscle strength and provide functional outcomes for people with neurological impairments [2–5]. Cycling for exercise, standing, stepping and walking are examples of exercise modes that may be induced via FES.

Fatigue is a natural phenomenon in voluntarily-activated muscles. Continuous or intermittent FES, although initially providing strong muscle activation, will also induce a subsequent decline of force [6]. This loss of force or torque normally occurs considerably more quickly in the muscles of individuals with SCI compared to those of able-bodied persons [7]. The progress of muscle fatigue however is more rapid and greater during FES, since the muscle fibres are activated synchronously and in “reverse recruitment order” by the stimulation pulses [8,9]. The rapid FES-induced muscle fatigue becomes particularly serious for neurological clinical populations when their paralyzed lower limbs muscles are activated to produce upright postures, because it may lead to unanticipated falls [10] or unexpected knee buckling [11]. The safety and efficacy of FES neuroprostheses systems must therefore rely upon their ability to reduce, quantify, or predict muscle fatigue during leg exercise [7]. One of the most established ways of monitoring evoked muscle activity and fatigue is using evoked electromyography signals (eEMG), otherwise known as m-waves.

M-waves are electrical nerve and muscle responses generated during electrically-evoked contractions. They represent the sum of all action potentials from the activated motor-units [12,13]. Variables extracted from m-waves have been considered to be potential proxies of muscle fatigue, since they appear to follow particular fatigue-related trends during FES exercise. M-wave amplitude variables are voltage levels detected at specific points in the m-waves, normally the peaks. They tend to decrease with muscle fatigue as motor-units stop firing, therefore not contributing to the summation of the action potentials [8]. The changes in m-wave duration and latency are mostly related to the decrease in fibre conduction velocity. These temporal variables on the other hand, tend to increase as muscle fatigue progresses [14]. Several authors have utilised the m-wave variables to characterize the “quality” of muscle contractions and the onset of fatigue in individuals with SCI [12,15]. In this paper, m-waves were adopted to monitor muscle fatigue in a specific FES-evoked training activity—–isometric knee extension.

FES interval training (*i.e.*, short bouts of FES-evoked leg exercise with recovery periods) has been proposed as an alternative approach to continuous walking for functional gait training in the SCI population [11], where muscle fatigue is of particular interest. The eEMG signal has been broadly investigated as a tool to assess muscle fatigue in applications under continuous and intermittent FES [16–19]. However, little is known about the effects of recovery periods between FES exercise sessions on eEMG responses.

In order to implement real-time strategies using m-waves to estimate muscle force and prevent knee buckling during FES, a greater understanding is required about the behaviour of m-waves under “real world” clinical conditions, which usually include recovery intervals between exercise or gait training sessions. It is crucial for the force or torque *versus* m-wave relationship to be constant throughout FES-evoked contractions, or if not constant, to be reliably modelled in order for the accurate muscle force production be derived from the m-waves for further control decisions. Therefore, this study sought to determine if the relationship between muscle torque and eEMG variables remained constant after ‘brief’ recovery periods (e.g., less than 60 s) following repeated intervals of isometric muscle training.

The main objectives were: (a) to quantify m-wave changes corresponding to decreasing torque elicited by progressive muscle fatigue; (b) to measure m-wave changes during the recovery of torque after the fatiguing muscle contractions; and (c) to assess the repeatability of the relationship m-wave × torque at different states of muscle fatigue.

## 2. Experimental Section

### 2.1. Subjects

Eight individuals with SCI volunteered to participate in this study. They were male, with complete or incomplete spinal cord lesions between C7 and T11, AIS A to C [20], aged 45.6 ± 15.7 y and 10.5 ± 6 y since their injury. Two subjects were AIS C with preserved voluntary motor function, and these individuals presented with stronger FES-induced muscle contractions, than the motor-complete (AIS A and B) subjects. According to our inclusion criteria, subjects were recruited if they presented: (a) with significant muscle paresis or complete paralysis of their lower limbs; (b) as responsive to electrical stimulation of the quadriceps muscle group; and, (c) possessed minimal spasticity elicited by FES. Subjects were excluded if they did not meet the inclusion criteria, or if they had sensorimotor ‘incomplete’ status with sufficient muscle strength that they could stand up and maintain stance through voluntary contractions of their own musculature. Subjects underwent at least ten 30 min FES cycling training sessions to provide sufficient muscle forces before their commencement of the FES-fatigue study. The study protocol was approved by the University Human Research Ethics Committee and written informed consent was completed prior to participation.

### 2.2. Instrumentation

Electrical stimulation was provided by a fully-configurable, constant-current laboratory neuromuscular electrical stimulator [21]. A commercial muscle dynamometer (Biodex Medical Systems, Shirley, NY, USA) was used to measure isometric torque evoked from the electrically stimulated quadriceps. A custom-made, computerized evoked EMG acquisition system (the UniSyd e^2^MG [22]) was utilized to control the stimulator and synchronize myoelectric signals with the torque outputs from the muscle dynamometer. All data was processed in real-time, and the instantaneous torque and m-wave were time-synchronised during experimental protocol.

### 2.3. Experimental Procedure

**Figure 1.**
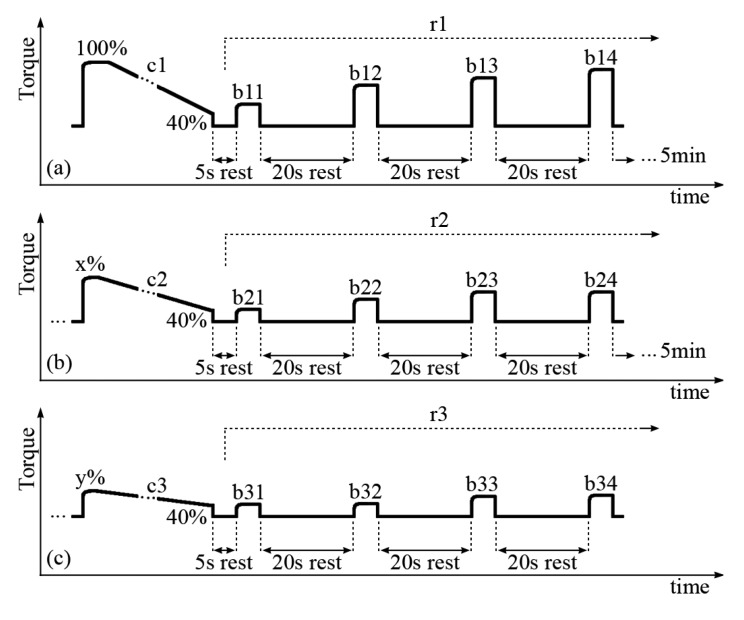
The experimental protocol deployed for data collection comprising three sets of contractions: (**a**) first contraction set; (**b**) second contraction set; and (**c**) third contraction set. Fatiguing contractions (a) c1, (b) c2 and (c) c3 were applied and ceased only when the torque declined to 40% of its initial maximum obtained during c1. Sequential bursts (b11,b12, *etc.*) were applied to assess the muscles during recovery intervals (r1, r2, *etc.*). The values x% (Figure 1b) and y% (Figure 1c) were empirical values of torque generated after the preceding recovery periods. Five min of rest were allowed between each set of contractions.

The subjects were seated in the dynamometer chair with one randomly selected leg attached to the dynamometer level arm at 60° (0° = full extension). The passive torque produced by the leg weight was measured and subtracted from all measurements (“gravity correction”). Brief isometric FES contractions were delivered to the quadriceps muscles while the pulse amplitude was gradually increased as to produce a torque of approximately 40 Nm, which is the average value deemed necessary to evoke FES stance [23–25]. This FES amplitude was set as constant for the entire trial, but was variable between SCI subjects in the range of 80–120 mA. A constant FES current amplitude was deployed, so that different muscle fibers would not be recruited between trials c1, c2 and c3 (described below). It was noted that different FES current amplitudes to achieve 40 Nm were achieved because there were variable degrees of FES-evoked muscle strength due to training status and/or level of lesion (e.g., two subjects were AIS C). Three sets of prolonged and short contractions were delivered to the quadriceps muscles during the test protocol. Each set was composed of a prolonged and fatiguing isometric muscle contraction, followed by four short FES bursts during the muscle recovery phase, as portrayed in Figure 1.

Each long contraction (denoted c1, c2 and c3) was applied until evoked torque fell to 40% of its initial maximum. When this lower limit was reached due to muscle fatigue, the stimulation was ceased for 5 s. Then, four short FES bursts (Figure 1, denoted b11, b12, b13 and b14; b21, b22, b23 and b24; b31, b32, b33 and b34) at the same current amplitude as the prolonged contraction were applied with 20s between them, within the three recovery periods (r1, r2 and r3). There was five min of rest-recovery between each set of long and short contractions which characteristically reached a smaller maximal peak torque during each set.

During c1, we measured the m-wave variables obtained from 100% to 40% of the maximal torque. During c2, the range was 70% to 40%, since not all the legs had recovered sufficiently to produce 80% of their maximal torque. For c3, variables were compared from 60% through 40% of the maximal torque produced during c1. The peak to peak amplitude (PtpA), and the m-wave area (Area) were normalized as percentages of their maximum values during c1: (*Var*/*Mix_c_*_1_)×100%. As the time between the stimulus artefact and the positive peak (PosT) presented an ascending slope with a decreasing torque, we normalized this variable using its minimal (unfatigued) value also obtained during c1: (*Var*/*Min_c_*_1_)×100%. This variable was then interpreted as the order of the m-wave positive peak delay as compared to its unfatigued (0%) state.

### 2.4. FES and eEMG Setup

The quadriceps muscles from both legs of each subject were investigated. Two medium size electrode pads (SE5240—Rectangle, 2″ × 3.5″—Empi DJO LLC., Vista, CA, USA) were positioned over the quadriceps muscle belly to produce isolated FES contractions of the rectus femoris (RF). This procedure was achieved by visual inspection and muscle palpation. FES parameters were set to biphasic pulses, frequency = 25 Hz, pulse width = 300 μs and amplitude up to 140 mA to attain the desired torque (at least 40 Nm). Before attaching the EMG electrodes, the skin was shaved and cleaned with alcohol swabs. All eEMG recording electrodes were disposable adhesive discs (ø = 20 mm), with solid Ag/AgCl gel (Meditrace™ Mini, Emergency Medical Products Inc., Cudahy, WI, USA). The active EMG electrode was positioned over the RF muscle belly, approximately 70 mm from the proximal stimulation electrode. The negative and reference eEMG electrodes were positioned over a non-active area, located more medially towards the hamstrings muscles. The monopolar arrangement was considered to superior to bipolar for this study, as it removed signal cancellation error and allowed genuine alterations in maximal m-wave magnitude to be observed [26,27]. However, the term “monopolar” has been employed only for convenience, since they were actually differential bipolar recordings with the negative and reference electrodes located over areas which were not producing action potentials.

### 2.5. eEMG Data Processing

**Figure 2.**
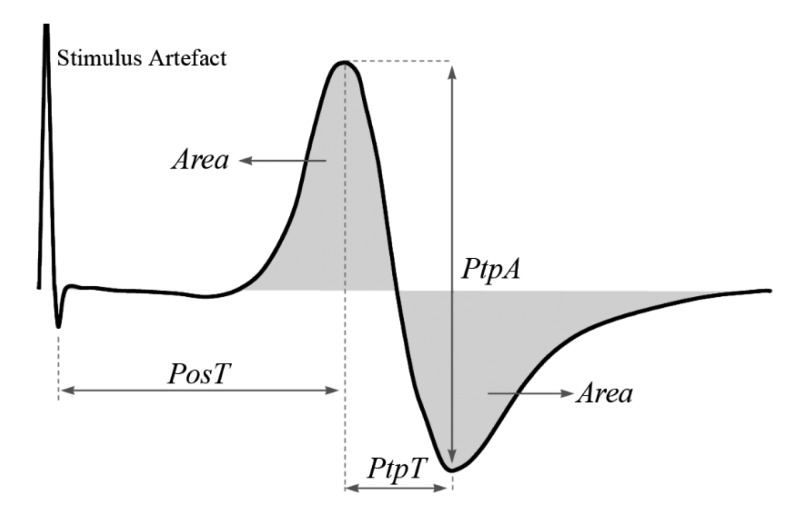
The m-wave and its real-time parameters: PosT: positive peak amplitude; NegT: negative peak amplitude; PtpA: peak-to-peak amplitude; PosT: positive peak time; NegT: negative peak time; PtP time: peak-to-peak time; and Area: m-wave area (sum of the two gray shaded areas).

The gain that was set to the instrumentation amplifier of the UniSyd e2MG system (INA126, Texas Instruments Inc., Dallas, TX, USA) varied from 1 to 5 times between subjects and legs. Band-pass Butterworth filter (4–480 Hz) was subsequently applied in the analogue signal conditioning, with an additional gain of 20 times and a variable DC voltage shift. The raw eEMG signal was then digitised at 4000 samples·s^−1^ by a 10-bit A/D converter. Each m-wave was processed in real-time, and only 3 predominant variables were extracted namely the peak to peak amplitude (PtpA), the time between the stimulus artefact and the positive peak (PosT), and the m-wave area (Area) as illustrated in Figure 2.

### 2.6. Statistical Analyses

The quardiceps muscle data from both limbs were pooled after analysing the PtpA extracted from the m-waves and verifying that there were no significant “between leg” effects between right and left limbs. All analyses were performed using SPSS statistical software (IBM SPSS Statistics, version 18, IBM Co., Austin, TX, USA), with the confidence level set to 5% (*p* ≤ 0.05).

**Figure 3.**
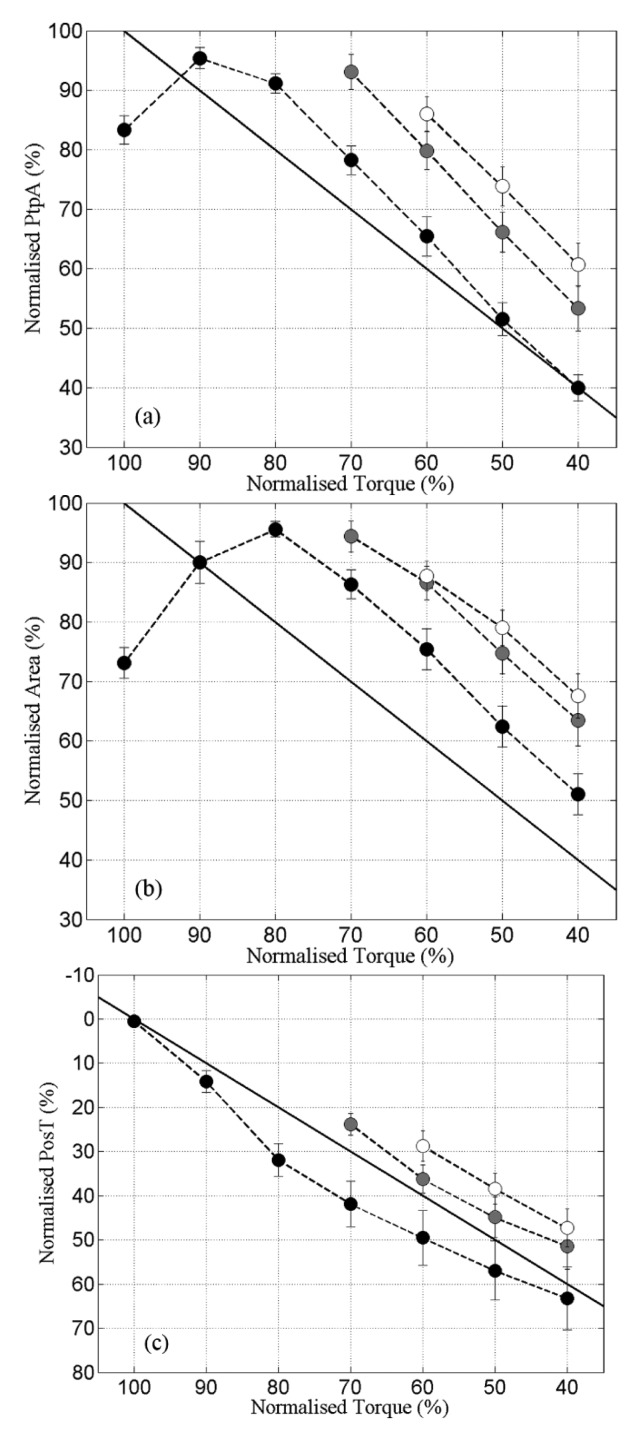
(**a**) PtpA × Torque; (**b**) Area × Torque and (**c**) PosT × Torque curves obtained during c1(●), c2 ( 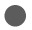) and c3 (○). Note that in (**c**) the y axis was intentionally inverted as PosT fatigued values were maximum, thus set as 100%. X axis represents normalised torque (%) reduces with time. The solid diagonal lines represent lines of identity. Data are mean ± standard errors.

The first data analysis briefly investigated the time-series curves of the m-wave variables. Our second analysis aimed to assess the repeatability of the relationship m-wave × torque through the three fatiguing contractions, when the thigh muscles became fatigued to a greater degree after each subsequent prolonged FES-induced contraction. The m-wave readings obtained at the same torque percentages during c1, c2 and c3 were compared (cf. Figure 3).

Two-way repeated measures ANOVAs were performed for each m-wave variable considering torque percentages and contractions as levels. One-way repeated measures ANOVAs were also performed at each percentage using contractions as levels only. Linear regressions (m-wave variables *versus* torque percentages) were fit to each leg's data, and one-way repeated measures ANOVA were conducted on the regression coefficients to identify if any differences existed between the m-wave-torque “slopes” during c1, c2 and c3. Pairwise *t*-tests were conducted (*p* ≤ 0.05) when the within-subjects main effect (Wilks' Lambda, λ) for m-wave “slopes” during long contractions (c1, c2, or c3) or recovery phases (r1, r2 and r3) was significant.

ANOVA was also performed on the on the linear regression coefficients (slopes) of the recovery periods r1, r2 and r3 torque-time relationship. In the text, “recovery” has been taken to mean change back towards initial values, not full restoration of initial torque. The degree of “shift” observed between the recovery lines were determined by the absolute difference between values extracted from bn1 and bn4, where *n* = 1 to 3 refers to the contraction set sequence (Figure 2). This provided information regarding the amount that m-wave variables recovered within each 60 s period.

## 3. Results

Eight male subjects with SCI were recruited for this study, with complete or incomplete spinal cord lesions between C7 and T11, AIS A to C [20] aged 45.6 ± 15.7 y and 10.5 ± 6 y since their injury incident.

### 3.1. Fatigue

After the first onset of neurostimulation during c1, while PtpA and Area curves were still increasing towards their maximal values, the muscle-evoked torque was already in decline for these SCI subjects. During this period, PtpA and Area, and the muscle torque shifted in opposite directions, as the time taken for Torque to reach its plateau gradually increased from c1 through c3. For PtpA and Area the opposite behaviour was observed-their peak values shifted towards the left side of the PtpA-time and Area-time curves from c1 to c3.

The relationships between PtpA, Area and PosT *versus* Torque during the 3 fatiguing contractions are portrayed in Figure 3. PtpA and Area during c2 were significantly higher than c1 at peak torque percentages ranging from 70%–40% of greatest torque. PosT fell consistently from c1 to c2, and between c2 and c3 although to a smaller degree (Figure 3c).

The linear regression coefficients (for “slopes”) showed no significant changes for PtpA (c1 = 1.29 ± 0.06; c2 = 1.33 ± 0.12; c3 = 1.27 ± 0.14; *p* > 0.05) or Area (c1 = 1.13 ± 0.079; c2 = 1.04 ± 0.15; c3 = 1.01 ± 0.18; *p* > 0.05). PosT displayed a small, but significant reduction, of slope between c1 and c3 only (c1 = −0.78 ± 0.10; c2 = −0.92 ± 0.15; c3 = −0.93 ± 0.13; *p* ≤ 0.05). The mean PtpA slopes during all contractions were steeper than the line of identity (*i.e.*, >1), indicating a faster change of PtpA than change of torque (Figure 3a). Area presented mean slopes very close to the line of identity (Figure 3b), and PosT changed at a slower pace (slope < 1) when compared with torque (Figure 3c). These observations based on slopes represented the mean differences between c1, c2 and c3 for the whole group. However, the pattern was not always consistent or uniform between subjects' muscles when analysed independently.

### 3.2. Recovery

**Figure 4.**
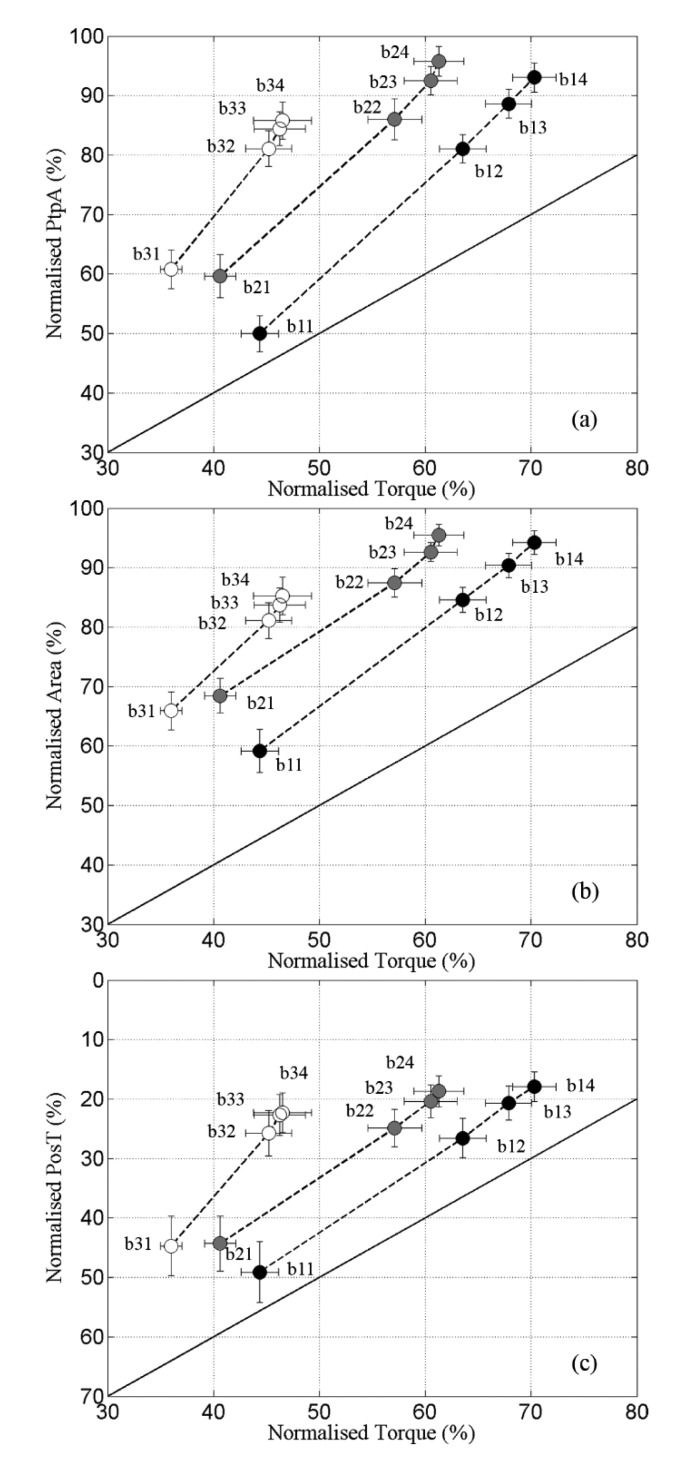
(**a**) *PtpA* × *Torque*; (**b**) *Area* × *Torque* and (**c**) *PosT* × *Torque* curves obtained during r1 (●), r2 ( 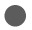) and r3 (○). Note that in (**c**) the y axis was intentionally inverted as *PosT* recovered towards 0% as opposed to 100% in (**a**) and (**b**). The solid diagonal lines represent lines of identity. Data are mean ± standard errors.

The recovery ratios between m-wave variables and torque are portrayed in Figure 4. The first novel finding was the strong linearity presented by all variables during r1, with a leg-averaged *R^2^* above 0.99 in all cases (Figure 4, markers denoted by ●).

Both m-waves and torque presented fast recovery during the first 20 s (b11–b12), which gradually reduced from b12 through b14. Still, the ratio *m-wave* × *torque* was fairly constant in r1with high *R^2^*. *PtpA* also maintained a tight linear relationship with *Torque* during r2, with *R^2^* between 0.96 and 0.99 for all legs. For *Area* and *PosT* this linearity was slightly affected towards the end of r2 (Figure 4b,c; b23 and b24, respectively), which produced decreased *R^2^* values in some legs (0.75 < R^2^ < 0.99 for *Area* and 0.86 < R^2^ < 0.99 for *PosT*). During c3, *R^2^* coefficients varied between 0.89–0.99, 0.76–0.99 and 0.82–0.99 for *PtpA*, *Area* and *PosT* respectively.

The regression coefficient (slopes) of *PtpA* were greater than the line of identity in all cases (r1 = 1.77 ± 0.20; r2 = 1.76 ± 0.13 and r3 = 2.36 ± 0.38), indicating that this variable always recovered at a faster rate than torque. No significant difference was found between the three recovery periods, which suggested a similar averaged ratio *PtpA* × *Torque* recovery. Slopes extracted from *Area* were also >1 during all recovery periods (r1 = 1.40 ± 0.16; r2 = 1.27 ± 0.15; r3 = 1.59 ± 0.38) with no significant difference between them. The results obtained for *PosT* were different, revealing a steeper negative slope during r3, and no significant difference between r2 and r1 (r1 = −1.42 ± 0.30; r2 = −1.32 ± 0.20; r3 = −3.11 ± 0.90). The recovery for *PosT* also happened faster than for the *Torque*.

Table 1 demonstrates that m-wave variables “recovered” to a larger extent than did muscle torque during r1–r3. *PtpA* showed recovery-ratios that were 1.66, 1.75 and 2.31 times larger than the *Torque* from r1 through r3. *PosT* was similar, with increasing recovery-ratios at 1.20, 1.24 and 2.07 larger than *Torque*. The m-wave *Area* always presented higher recovery than *Torque*, but not as much increase on the ratios through the contractions as *PtpA* and *PosT* (1.35, 1.31 and 1.79).

## 4. Discussion

Evoked electromyography signals (eEMG; m-waves) have previously been proposed as a sensor for muscle forces [12,15], where the latter might be impractical to measure in the clinical environment or where FES-evoked muscle training does not have a proxy for force (e.g., during upright FES-evoked gait training). Successful attempt were made by Zhang *et al.*, in predicting the FES-induced torque using eEMG signal in individuals with SCI [28]. This study sought to determine if the relationship between muscle torque and eEMG variables remained constant after ‘brief’ recovery periods (e.g., less than 60 s), following repeated intervals of isometric muscle training, as is commonly practiced in the clinical milieu. In this study, 48 FES-induced muscle contractions were evoked in both leg muscles of eight SCI subjects with ‘complete’ or ‘incomplete’ spinal cord lesions between C7 and T11.

From the beginning of the first fatiguing contraction (c1), PtpA-time and Area-time curves increased gradually to reach their respective plateaus when the evoked-muscle torque was already in decline. This phenomenon, called “m-wave potentiation”, has been identified in prior investigations [15,19,29], and has been suggested as elicited by FES-induced increases in sodium-potassium pump activities. In the present study, m-wave potentiation always preceded force decay, as exhibited by these two m-wave variables during fatigue. The duration of the m-wave potentiation period was reduced during c2 and even further shortened during c3, when the muscles were more fatigued. Conversely, torque curves presented longer rising time to plateau during c2 and c3, preceding their subsequent decays.

This pattern suggested that during a brief initial period during the muscle contractions, the decay of torque could have been caused by events other than motor unit activation failure (which are eEMG detectable). Although not fully understood in the SCI population (discussed in [30]), possible metabolic events eliciting fatigue during FES-evoked contractions include: (i) shortage of fuel (substrates) within the muscle fiber, and/or; (ii) accumulation of substances (metabolites) within the muscle fiber, which interfere either with the release of calcium (Ca^2+^) or with the ability of calcium to stimulate muscle contraction. Relationships between PtpA and torque (or Area and torque) could be identified only after these m-wave variables began to decline. We propose that following the m-wave potentiation period, a decline in PtpA and Area represented a reduced number of motor-units producing FES-synchronized action potentials. This, in turn, could have reduced torque production—both peak torque and time-to-peak-torque. PosT-time curves increased after stimulation onset and throughout the whole contraction periods. These changes could have been attributed to: (i) a decrease in fibre conduction velocity; (ii) an increase in action potential latency; and/or, (iii) a selective drop out of the fast motor-units [12]. These factors also might explain the pattern of reduced torque production during the fatiguing contractions, c1–c3.

By comparing c1 against c2 and c3, we sought to verify whether these physiological factors might influence m-waves in a similar manner at the three different stages of fatigue separated by short recovery periods, given that the same torque was being produced. Our findings revealed dissimilar m-wave patterns during each subsequent fatiguing contraction. This finding implied that advanced signal processing and control techniques are required to use the m-wave signals as direct proxies of muscle torque. It was also identified that the fatigue ratios between m-wave variables and torque were mostly unchanged from c1 through c3, as demonstrated by the unchanged slopes of PtpA × Torque and Area × Torque relationships.

PtpA changes detected during each fatiguing contraction tended to occur at a faster rate when compared with Torque, while PosT changes were always slower. The m-wave Area is a computed variable involving both m-wave amplitude and duration. The m-wave duration also tends to increase with fatigue [19,31], in a similar manner as observed for PosT. The Area therefore presented alterations which were fatigue-affected both by decreases in amplitude and increases in duration, producing magnitude generally between PtpA and PosT.

The recovery analysis indicated that both the torque and m-wave variables recovered most quickly during the first 20 s of recovery. In fact, only minor recovery was detected in intervals between the fourth bursts and the subsequent long contractions (b14 to c2 and b24 to c3, Figure 1). An intriguing observation was the fact that different torque readings were obtained during b11, b21 and b31, with mean values of 44%, 41% and 36% of highest torque, respectively. All fatiguing contractions preceding their respective recovery intervals were automatically ceased when the torque reached the 40% de-limited torque value. This means that the torque changed in different ways within the 5 s intervals separating c1, c2 and c3 from b11, b21 and b31, depending on how fatigued the muscles were. Between c1 and b11, a brief recovery took place which slightly increased the torque. Within c2–b21 and c3–b31 the torque remained relatively unchanged and decreased respectively.

In general, the slopes obtained during r1 for all m-wave variables presented no significant difference when compared to r2. This implied that the averaged recovery ratios m-wave × torque were similar through r1 and r2. However, this ratio was always favourable to the m-wave variables indicating faster recovery than the torque. The slopes during r3 were slightly steeper (mostly non-significant) than in r1 and r2. This result associated with the low torque found on b34 suggested that the capacity of torque recovery was more affected than the m-wave capacity of recovery, as the muscles became more fatigued.

Even though we did not focus the data analyses on the time duration of each contraction, we could still observe a high variability in the time to exhaustion (set as duration until 40% of the maximal torque) among subjects and also between legs within the same individual. This problem was circumvented as we designed our protocol to compare m-wave readings at specific torque percentages, and not at specific time readings during the contractions.

In clinical FES applications, such as during FES-induced standing or walking, where direct measures of muscle torque measurement are impractical, these temporal variations within and between m-waves limit their use as a proxy for force or a predictor of fatigue-failure. If an effective lower-limb FES feedback system based on m-waves is achievable, equations designed for individuals or even individual legs are more likely to succeed. Yet, these potential models need to find a way for compensating alterations in the m-wave × torque relationship should rest intervals be allowed between exercise sessions.

Another limitation for the ability of m-waves to be used as a proxy for muscle fatigue during FES-evoked contractions, was the relatively modest sample size (*n* = 8) used in the current study and the potential for large intra-individual difference of m-wave characteristics between subjects. The issue of modest sample size is a common limitation for studies that have employed individuals with spinal cord injury, and our ‘opportunistic’ sample of eight subjects was in line with other investigations in this population. To reduce intra-individual differences, future research should investigate how differences of m-wave characteristics might be “normalized” between subjects, or whether there is some inherent “threshold” below which muscle fatigue-failure occurs during functional activities such as FES-evoked standing or FES-induced gait.

## 5. Conclusions

Different m-wave readings were detected when the same muscle torque was being generated, depending upon how fatigued the quadriceps muscles were. A constant factor between the change rates of m-waves and torque during both fatiguing contractions and recovery intervals appeared to exist. Nonetheless, variations found on absolute values before and after different degrees of fatigue did clarify this dynamics. The results presented in this paper revealed that brief recovery intervals provided during FES exercise sessions performed by individuals with SCI could drastically interfere with any attempt of using m-waves as a direct proxy for torque estimation. The instantaneous pre-state of muscle fatigue at the moment of the FES-evoked exercise could also present as a confounding issue. The potential utility of m-waves being a sensor of muscle fatigue during FES-evoked physical activities must be further investigated to gain better understanding of the mechanisms of muscle fatigue and recovery in this population with long-standing muscle paresis/paralysis.

## Figures and Tables

**Table 1.** Recovery percentage (*mean* ± *SE*) within first 60s (r1 > r2 > r3, *p* < 0.05).

**Variable**	**r1 (b14**–**b11)**	**r2 (b24**–**b21)**	**r3 (b34**–**b31)**
Torque (%)	25.94 ± 2.45	20.65 ± 2.03	10.82 ± 2.13
PtpA (%)	43.06 ± 3.25	36.12 ± 3.25	24.98 ± 3.44
Area (%)	35.10 ± 3.73	27.01 ± 3.44	19.34 ± 4.10
PosT (%)	31.16 ± 3.34	25.61 ± 2.68	22.42 ± 2.76

## References

[1] Mizrahi J. (1997). Fatigue in muscles activated by functional electrical stimulation. Crit. Rev. Phys. Rehabil. Med..

[2] Davis G.M., Hamzaid N.A., Fornusek C. (2008). Cardiorespiratory, metabolic, and biomechanical responses during functional electrical stimulation leg exercise: Health and fitness benefits. Artif. Organs.

[3] Hamzaid N.A., Davis G.M. (2009). Health and Fitness Benefits of Functional Electrical Stimulation-Evoked Leg Exercise for Spinal Cord–Injured Individuals. Top. Spinal Cord Injury Rehabil..

[4] Philips C.A. (1991). Functional Electrical Stimulation: Technological Restoration after Spinal Cord Injury.

[5] Ragnarsson K.T. (2008). Functional electrical stimulation after spinal cord injury: Current use, therapeutic effects and future directions. Spinal Cord.

[6] Heasman J.M., Scott T.R.D., Vare V.A., Flynn R.Y., Gschwind C.R., Middleton J.W., Butkowski S.B. (2000). Detection of fatigue in the isometric electrical activation of paralyzed hand muscles of persons with tetraplegia. IEEE Trans. Rehabil. Eng..

[7] Ibitoye M.O., Estigoni E.H., Hamzaid N.A., Abdul Wahab A.K., Davis G.M. (2014). The Effectiveness of FES-Evoked EMG Potentials to Assess Muscle Force and Fatigue in Individuals with Spinal Cord Injury. Sensors.

[8] Bigland-Ritchie B., Jones D.A., Woods J.J. (1979). Excitation frequency and muscle fatigue: Electrical responses during human voluntary and stimulated contractions. Exp. Neurol..

[9] Karu Z.Z., Durfee W.K., Barzilai A.M. (1995). Reducing muscle fatigue in FES applications by stimulating with N-let pulse trains. IEEE Trans. Biomed. Eng..

[10] Rushton D.N. (1997). Functional electrical stimulation. Physiol. Measur..

[11] Crosbie J., Russold M., Raymond J., Middleton J.W., Davis G.M. (2009). Functional Electrical Stimulation-Supported Interval Training Following Sensorimotor-Complete Spinal Cord Injury: A Case Series. Neuromodul.: Technol. Neur. Interf..

[12] Chang Y.J., Shields R.K. (2002). Within-train neuromuscular propagation varies with torque in paralyzed human muscle. Muscle Nerve.

[13] Merletti R., Knaflitz M., de Luca C.J. (1992). Electrically evoked myoelectric signals. Crit. Rev. Biomed. Eng..

[14] Merletti R., Knaflitz M., de Luca C.J. (1990). Myoelectric manifestations of fatigue in voluntary and electrically elicited contractions. J. Appl. Physiol..

[15] Tepavac D., Schwirtlich L. (1997). Detection and prediction of FES-induced fatigue. J. Electromyogr. Kinesiol..

[16] Chesler N.C., Durfee W.K. (1997). Surface EMG as a fatigue indicator during FES-induced isometric muscle contractions. J. Electromyogr. Kinesiol..

[17] Griffin L., Jun B.G., Covington C., Doucet B.M. (2008). Force output during fatigue with progressively increasing stimulation frequency. J. Electromyogr. Kinesiol..

[18] Mizrahi J., Levin O., Aviram A., Isakov E., Susak Z. (1997). Muscle fatigue in interrupted stimulation: Effect of partial recovery on force and EMG dynamics. J. Electromyogr. Kinesiol..

[19] Yu N.-Y., Chen J.J., Ju M.-S. (1999). Study of the electrically evoked EMG and torque output during the muscle fatigue process in FES-induced static and dynamic contractions. BAM-PADOVA.

[20] Marino R.J., Barros T., Biering-Sorensen F., Burns S.P., Donovan W.H., Graves D.E., Haak M., Hudson L.M., Priebe M.M. (2003). International standards for neurological classification of spinal cord injury. J. Spinal Cord Med..

[21] Fornusek C., Davis G.M., Sinclair P.J., Milthorpe B. (2004). Development of an Isokinetic Functional Electrical Stimulation Cycle Ergometer. Neuromodulation.

[22] Estigoni E.H., Fornusek C., Song T., Davis G.M. A novel system for evoked EMG analysis: Design, construction, and validation.

[23] Bajd T., Kralj A., Turk R. (1982). Standing-up of a healthy subject and a paraplegic patient. J. Biomech..

[24] Davis J.A., Triolo R.J., Uhlir J., Bieri C., Rohde L., Lissy D., Kukke S. (2001). Preliminary performance of a surgically implanted neuroprosthesis for standing and transfers—Where do we stand?. J. Rehabil. Res. Dev..

[25] Kotake T., Dohi N., Kajiwara T., Sumi N., Koyama Y., Miura T. (1993). An analysis of sit-to-stand movements. Arch. Phys. Med. Rehabil..

[26] Pierrot-Deseilligny E., Mazevet D. (2000). The monosynaptic reflex: A tool to investigate motor control in humans. Interest and limits. Neurophysiol. Clin..

[27] Tucker K.J., Türker K.S. (2005). A new method to estimate signal cancellation in the human maximal m-wave. J. Neurosci. Methods.

[28] Zhang Q., Hayashibe M., Fraisse P., Guiraud D. (2011). FES-induced torque prediction with evoked EMG sensing for muscle fatigue tracking. IEEE/ASME Trans. Mech..

[29] Indurthy M., Griffin L. (2007). Effect of random interpulse interval modulation on neuromuscular fatigue. Muscle Nerve.

[30] Kesar T., Binder-Macleod S. (2006). Effect of frequency and pulse duration on human muscle fatigue during repetitive electrical stimulation. Exp. Physiol..

[31] Rabischong E. (1996). Surface action potentials related to torque output in paraplegics' electrically stimulated quadriceps muscle. Med. Eng. Phys..

